# Ultrasensitive biosensing platform for *Mycobacterium tuberculosis* detection based on functionalized graphene devices

**DOI:** 10.3389/fbioe.2023.1313494

**Published:** 2023-12-20

**Authors:** Giwan Seo, Geonhee Lee, Wooyoung Kim, Inyoung An, Myungwoo Choi, Sojeong Jang, Yeon-Joon Park, Jeong-O. Lee, Donghwi Cho, Edmond Changkyun Park

**Affiliations:** ^1^ Research Center for Bioconvergence Analysis, Korea Basic Science Institute, Cheongju, Republic of Korea; ^2^ Critical Diseases Diagnostics Convergence Research Center, Korea Research Institute of Bioscience and Biotechnology, Daejeon, Republic of Korea; ^3^ Advanced Materials Division, Korea Research Institute of Chemical Technology, Daejeon, Republic of Korea; ^4^ Department of Materials Science and Engineering, Korea University, Seoul, Republic of Korea; ^5^ Department of Laboratory Medicine, Seoul St. Mary’s Hospital, The Catholic University of Korea, Seoul, Republic of Korea

**Keywords:** graphene, field-effect transistor (FET), *Mycobacterium tuberculosis*, MPT64, biosensor

## Abstract

Tuberculosis (TB) has high morbidity as a chronic infectious disease transmitted mainly through the respiratory tract. However, the conventional diagnosis methods for TB are time-consuming and require specialists, making the diagnosis of TB with point-of-care (POC) detection difficult. Here, we developed a graphene-based field-effect transistor (GFET) biosensor for detecting the MPT64 protein of *Mycobacterium tuberculosis* with high sensitivity as a POC detection platform for TB. For effective conjugation of antibodies, the graphene channels of the GFET were functionalized by immobilizing 1,5-diaminonaphthalene (1,5-DAN) and glutaraldehyde linker molecules onto the graphene surface. The successful immobilization of linker molecules with spatial uniformity on the graphene surface and subsequent antibody conjugation were confirmed by Raman spectroscopy and X-ray photoelectron spectroscopy. The GFET functionalized with MPT64 antibodies showed MPT64 detection with a detection limit of 1 fg/mL in real-time, indicating that the GFET biosensor is highly sensitive. Compared to rapid detection tests (RDT) and enzyme-linked immunosorbent assays, the GFET biosensor platform developed in this study showed much higher sensitivity but much smaller dynamic range. Due to its high sensitivity, the GFET biosensor platform can bridge the gap between time-consuming molecular diagnostics and low-sensitivity RDT, potentially aiding in early detection or management of relapses in infectious diseases.

## 1 Introduction

Two-dimensional (2D) materials have strong potential as biosensor platforms with low detection limits (Bolotsky et al., 2019; Rohaizad et al., 2021; Asif et al., 2022; Chen et al., 2022; Amara et al., 2023; Vaghasiya et al., 2023). Graphene, a 2D sheet of hexagonally arranged carbon atoms, is a promising candidate 2D material for use in biosensors, where it directly contacts its surroundings and reacts to electrostatic variations (Szunerits and Boukherroub, 2018; Sengupta and Hussain, 2021). Indeed, graphene has been demonstrated to be a promising sensing material not only for chemical sensors but also for biomolecular sensors, including sensors for infectious disease detection (Pumera, 2011; Justino et al., 2017; Cao et al., 2020). Although graphene is a semi-metal without a bandgap, its high carrier mobility, large specific surface area, chemical inertness, and low-noise properties make it a promising candidate for use in biosensors.

Recently, various 2D materials such as 2D carbides and nitrides of transition metals (MXene) and molybdenum disulfide (MoS2) etc. Have been utilized to fabricate biosensors, showcasing their potential for sensor applications (Agarwal and Chatterjee, 2018; Chaudhary et al., 2023; Sen et al., 2023). While these diverse 2D materials have demonstrated certain potential as sensor materials, it is still in the early stages of research, and more results need to be accumulated. In contrast, graphene is a widely used sensor material due to its well-established industrial scale growth methods and commercial availability (Zhu et al., 2018). Since graphene does not have a bandgap, on/off ratio of Graphene-based field-effect transistors (GFETs) is relatively small compared to that of other semiconductor materials. However, low noise characteristic of GFET may compensate for small on/off ratio of the material (Fu et al., 2017). Also, functionalization of graphene surfaces is easier and straightforward compared to other semiconductor materials. While linkers bind via π-stacking do not alter electronic properties of graphene significantly, surface functionalization or change of contact materials affect greatly on semiconductor low dimensional materials (Wang et al., 2019; Azadmanjiri et al., 2020; Wang and Chhowalla, 2021). Field-effect transistor (FET)-based biosensors offer the advantage of utilizing semiconductor production processes for sensor fabrication when compared to other electrochemical sensors. This allows for mass production of sensors, which can be effectively employed for mass determination of infection status, aligning with the ultimate goal of point-of-care testing (POCT).

Airborne respiratory infectious diseases continue to emerge frequently and to rapidly disseminate across the globe. One such example is tuberculosis (TB), a chronic infectious disease with a substantial prevalence and mortality rate, primarily transmitted through the respiratory route. *Mycobacterium tuberculosis* (*M. tuberculosis,* MTB) has posed significant challenges to healthcare systems worldwide and has become a threat to public health (Chai et al., 2018; Russell, 2021). On a global scale, MTB has affected more than 2.5 billion individuals, with approximately 10 million new cases emerging annually, contributing to a worldwide TB pandemic. The critical factor for TB screening relies on rapid biomarker-driven tests to ensure precise diagnosis and early treatment. The current TB diagnosis typically involves culturing bacteria from suspected patient samples and then confirming the diagnosis using rapid antigen detection kits or molecular diagnostics (Gupta and Kakkar, 2018). In addition, MTB bacterial culture tests are important not only for diagnosing TB but also for assessing patient recovery and determining drug susceptibility. However, bacterial culture tests require an incubation period that is often too long for rapid antigen detection kits to be used effectively. Therefore, a need exists for an antigen detection method that does not require a culture process and is much more sensitive than the currently used diagnostic methods.

Herein, we demonstrate GFETs for detecting the MPT64 antigen using the anti-MTP64 antibody (MPT64 Ab) as a receptor. MPT64 is a well-known diagnostic antigen protein for the detection of MTB. Commercially available rapid antigen diagnostic tests and ELISA kits for TB use MPT64 as a detection target. To conjugate the MPT64 Ab on the sensing area (i.e., the graphene surface), graphene-based sensing devices are modified with efficient interface coupling agents: 1,5-diaminonaphthalene (1,5-DAN) and glutaraldehyde (GA). Raman spectroscopy and X-ray photoelectron spectroscopy (XPS) analyses provide a structural fingerprint of the 1,5-DAN-treated graphene surface. In addition, the surface roughness of graphene is found to increase after chemical bonding because of the attachment of MPT64 Ab. Structural changes on the graphene surface due to efficient antibody–antigen interactions make graphene a material with good sensing performance for the rapid and sensitive detection of the MPT64 antigen.

## 2 Materials and methods

### 2.1 Sample preparation

The specific uses for the reagent listed in Supplementary Table S1. Graphene was grown on Cu foil (99.8%, Alfa Aesar, Ward Hill, MA, United States) by chemical vapor deposition (CVD). Detailed synthesis methods are described elsewhere (Jeong et al., 2022). Graphene was transferred onto SiO_2_/Si substrates using the conventional wet transfer method (Lee et al., 2023). The graphene/Cu foil surface was coated with polymethyl methacrylate (PMMA) C4 solution (Microchem, Newton, MA, United States) in a two-step spin-coating process with rotation speeds of 500 rpm for 10 s and 3,000 rpm for 30 s. The Cu foil was then etched with CE-100 Cu etchant (Transene, Danver, MA, United States). After the Cu foil was etched, the PMMA/graphene layer was rinsed with deionized (DI) water to remove residual Cu etchant. This layer was then transferred to a SiO_2_/Si substrate and dried overnight at room temperature. Finally, the PMMA layer was removed by immersing the PMMA-coated substrate in acetone for 2 h, rinsed with isopropyl alcohol, and dried with blown N_2_.

### 2.2 Fabrication of a graphene-based biosensor platform

The graphene biosensor platform was fabricated using conventional photolithography processes. First, AZ5214 photoresist (PR) was spin-coated in two steps—at 1,000 rpm for 10 s and then at 4,000 rpm for 30 s—onto the surface of graphene and heated at 90°C for 3 min. The samples were then exposed to UV light (i-line) for 5 s using a mask aligner (MDA-4000, Midas Systems, Deajeon, Korea) and developed in 300MIF developer for 1 min. The graphene area without PR patterns was removed using a plasma system (200LF plasma processing system, Femto Science, Hwaseong, Korea) with O_2_ (50 sccm) and Ar (20 sccm) plasma generated at 80 W; the plasma processing was conducted for 30 min Finally, the remaining PR layer was removed in an acetone solution for 1 h and rinsed with isopropyl alcohol (IPA). The length (*L*) and width (*W*) of the graphene channel were 2,000 × 100 μm^2^, respectively. The fabricated graphene-based device was covered with a passivation film to reduce the interference signal during electrical measurements.

Next, the contact electrodes were deposited onto the patterned graphene sample covered with a patterned stencil mask using a thermal evaporation system with Au (50 nm) and Cr (1 nm). Finally, a polyethylene terephthalate (PET) adhesive film with a 5 mm diameter hole was attached to the surface of the graphene device as a passivation layer to protect areas outside the channel layer from the surrounding solvents.

### 2.3 Surface modification of graphene and MPT64 antibody conjugation

The fabricated graphene-based device was soaked in a 1 mM solution of 1,5-DAN (Sigma-Aldrich, Bulington, MA, United States) in methanol for 1 h at room temperature and then dipped in a 2% (v/v) solution of GA (Sigma-Aldrich) in phosphate-buffered saline (PBS) in a humid environment for another 6 h. The modified graphene-based device was rinsed several times with PBS and DI water. Finally, the modified device was exposed to 200 μg/mL of MPT64 Ab (Cat. No. ab193435; Abcam, Cambridge, United Kingdom) overnight and washed with PBS and DI water.

### 2.4 Measuring the sensing performance of the graphene-based biosensor

The electrical performance of the biosensor was evaluated using a semiconductor analyzer (2634B, Keithley, Solon, OH, United States) and a probe station. A drain–source bias voltage of ∼10 mV was applied in a real-time measurement. The detected electrical response signal was normalized as [Δ*I*/*I*_0_] = (*I* − *I*_0_)/*I*_0_, where *I* is the detected real-time current and *I*_0_ is the initial current.

### 2.5 Characterization

The surface roughness due to the functionalization of graphene with MPT64 Ab and 1,5-DAN was analyzed using atomic force microscopy (AFM) (Digital Instruments Dimension 3,000, Veeco, Plainview, NY, United States). A contact-angle measurement system (optical system, SPVT-2000) was used to analyze the surface tension of graphene functionalized with MTP64 Ab and 1,5-DAN. Chemical binding information for the graphene surface was acquired by XPS (K-Alpha XPS system, ThermoFisher Scientific, MA, United States) with incident beams generated by an Al X-ray source (*hν* = 1,486.6 eV) and a pass energy of 50 eV. UV–Vis spectrophotometry (UV-2550, Shimadzu, Kyoto, Japan) was used to study the optical properties of MTP64 Ab-conjugated and 1,5-DAN-functionalized graphene on quartz substrates. Raman spectra and mapping analysis of the graphene surface were conducted using a Raman system (InVia, Renishaw, Derbyshire, United Kingdom) to confirm the uniformity of the MTP64 Ab and 1,5-DAN functionalization. Large-area Raman mapping images were created from 196 spectra (30 × 30 μm^2^ area) with a 2.5 μm measurement spacing in both the horizontal and vertical directions.

### 2.6 Rapid diagnostic test kit

For the sensitivity test using an RDT kit (Abbott, Chicago, IL United States), 100 μL of MPT64 antigen proteins was applied to the round hole of the RDT kit. After 15 min, the intensity of the test (positive for MPT64 antigen) and control lines was confirmed. All tested MPT64 proteins were prepared at 1 μg/mL in PBS solution (0.1 M, pH 7.4) and serially diluted at 1:10 with PBS.

### 2.7 Enzyme-linked immunosorbent assay

Immunoplates (Cat. No. 439454, ThermoFisher Scientific) were coated overnight at 4°C with various amounts (1,000, 100, 10, 1, 0.1, 0.01, 0.001, 0.0001, and 0 ng/mL) of MTB protein (MPT64, Cat. No. AB225589; Abcam) in 100 μL of 0.05 M carbonate–bicarbonate buffer (Cat. No. C3041-100CAP; Sigma-Aldrich, MO, United States) per well. After extensive washing with PBS containing Tween 20 (PBST), the plates were blocked for 1 h with 5% bovine serum albumin (BSA) in PBS with 0.1% Tween-20Subsequently, a 1:1,000 diluted MTP64 Ab (Cat. No. AB193435; Abcam) in 5% BSA in PBST was added to each well and incubated for 1 h. Following another round of extensive washing with PBST, the bound antibodies were incubated with horseradish peroxidase (HRP)-conjugated anti-rabbit IgG (1:5,000; Cat. No. #7074; Cell Signaling Technology, MA, United States) detection antibody for 1 h. After thorough washing with a wash buffer, stabilized chromogen (TMB solution, Cat. No. 34028; ThermoFisher Scientific) was added. The immunoplates were allowed to react for 10 min, and the reaction was stopped by adding 2 N H_2_SO_4_. The optical density (OD) value was measured at 450 and 650 nm using a microplate reader (SpectraMax, Molecular Devices, San Jose, CA, United States).

## 3 Results

### 3.1 Surface functionalization on graphene for TB sensing

**FIGURE 1:**
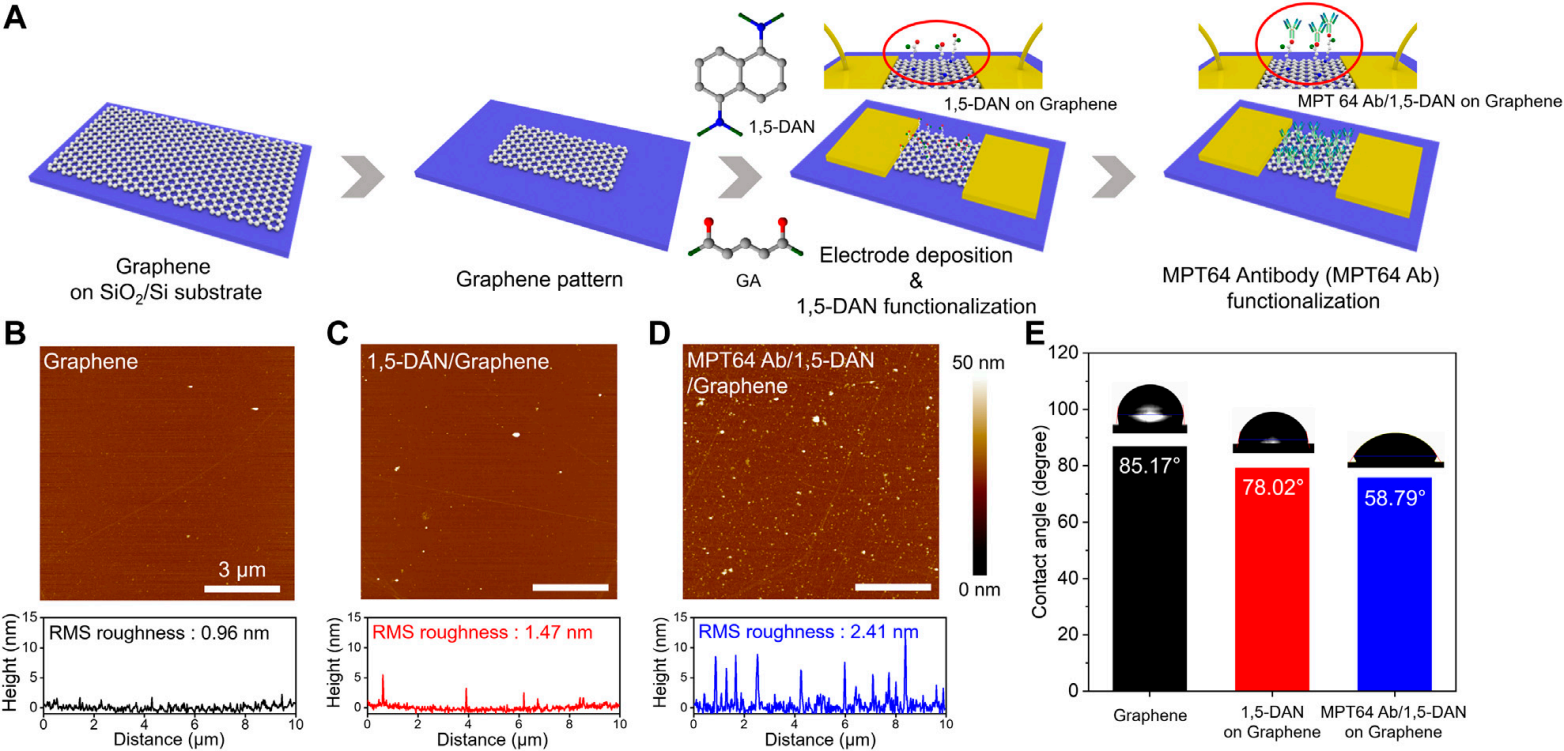
Functionalization of the graphene surface for the TB sensing device **(A)**. Schematic of the surface modification and MPT64 antibody (MTP64 Ab) conjugation process with 1,5-DAN and GA for the graphene-based sensing device **(B–D)**. AFM images of the pristine graphene (i.e., before surface modification), the graphene after 1,5-DAN treatment, and the graphene after MTP64 Ab conjugation (i.e., MTP64 Ab attachment onto the 1,5-DAN treated graphene surface), respectively. The bottom graphs correspond to the height profiles in the corresponding three AFM images and to our interpretation of the attachment of the MTP64 Ab after the surface functionalization. The scale bar in the image is 3 mm **(E)**. Contact angle analyses of pristine graphene, 1,5-DAN-modified graphene, and MTP64 Ab-conjugated graphene.

Figure 1A shows an overview of the functionalization of a graphene surface with MTP64 Ab to develop a graphene-based biosensor platform for TB diagnosis. The sensing probe material (MTP64 Ab) of the biosensor was functionalized on a well-defined graphene layer; details of the fabrication processes are described in the Experimental section. In the initial stage, the graphene surface becomes functionalized with 1,5-DAN, which effectively binds to the graphene surface through π–π interactions between the phenyl groups of 1,5-DAN and the graphene surface. Subsequently, GA, composed of two aldehyde (–CHO) groups, functions as an intermediate cross-linker via Schiff base reaction mechanisms, binding 1,5-DAN and MTP64 Ab (Fabbrizzi, 2020). In the first step of the GA treatment, one of the aldehyde groups formed a chemical bond with the amine (–NH_2_) groups of 1,5-DAN. Both 1,5-DAN and GA are efficient interface coupling agents that immobilize the antibody onto the graphene surface (Zhang et al., 2012; Goyal and Shim, 2015). In the second step, the other aldehyde group of GA is attached to the amine group of the antibody via a reaction mechanism similar to the first step (Bahmanyar and Houk, 2001). Therefore, through the previously described reaction steps, functionalization of graphene with 1,5-DAN and GA effectively captures TB antibodies (MTP64 Ab) on the graphene surface, enabling effective TB diagnosis in graphene-based biosensor measurements.

We used AFM (Figures 1B–D; Supplementary Figure S1) to observe the surface properties of the graphene and functionalized graphene samples. Initially, pristine graphene exhibited a root mean square (RMS) roughness of 0.96 nm, indicating an atomically smooth surface (Seo et al., 2020). After graphene was functionalized with 1,5-DAN and MTP64 Ab/1,5-DAN, the measured RMS roughness values were 1.47 and 2.41 nm, respectively. This change in surface conditions indicates the successful attachment of the probe material onto the graphene. Figure 1E illustrates the contact-angle analysis based on the functionalization of the graphene surface. Pristine graphene exhibited hydrophobic behavior, with a contact angle of approximately 85°. After material functionalization, the graphene surface gradually became hydrophilic (∼78° for 1,5-DAN and ∼59° for MTP64 Ab/1,5-DAN). The 1,5-DAN layer is generally hydrophobic because of its aromatic amine functional groups (Alzate-Carvajal et al., 2018); however, it became hydrophilic after the reaction with GA (Lim et al., 2013; Lo et al., 2020). In addition, the inclusion of MTP64 Ab with a hydrophilic amino acid led to enhanced surface hydrophilicity of the functionalized graphene surface (Sypabekova et al., 2017), supported by the surface analysis and contact-angle analysis.

### 3.2 Raman spectroscopy-based surface analysis

**FIGURE 2:**
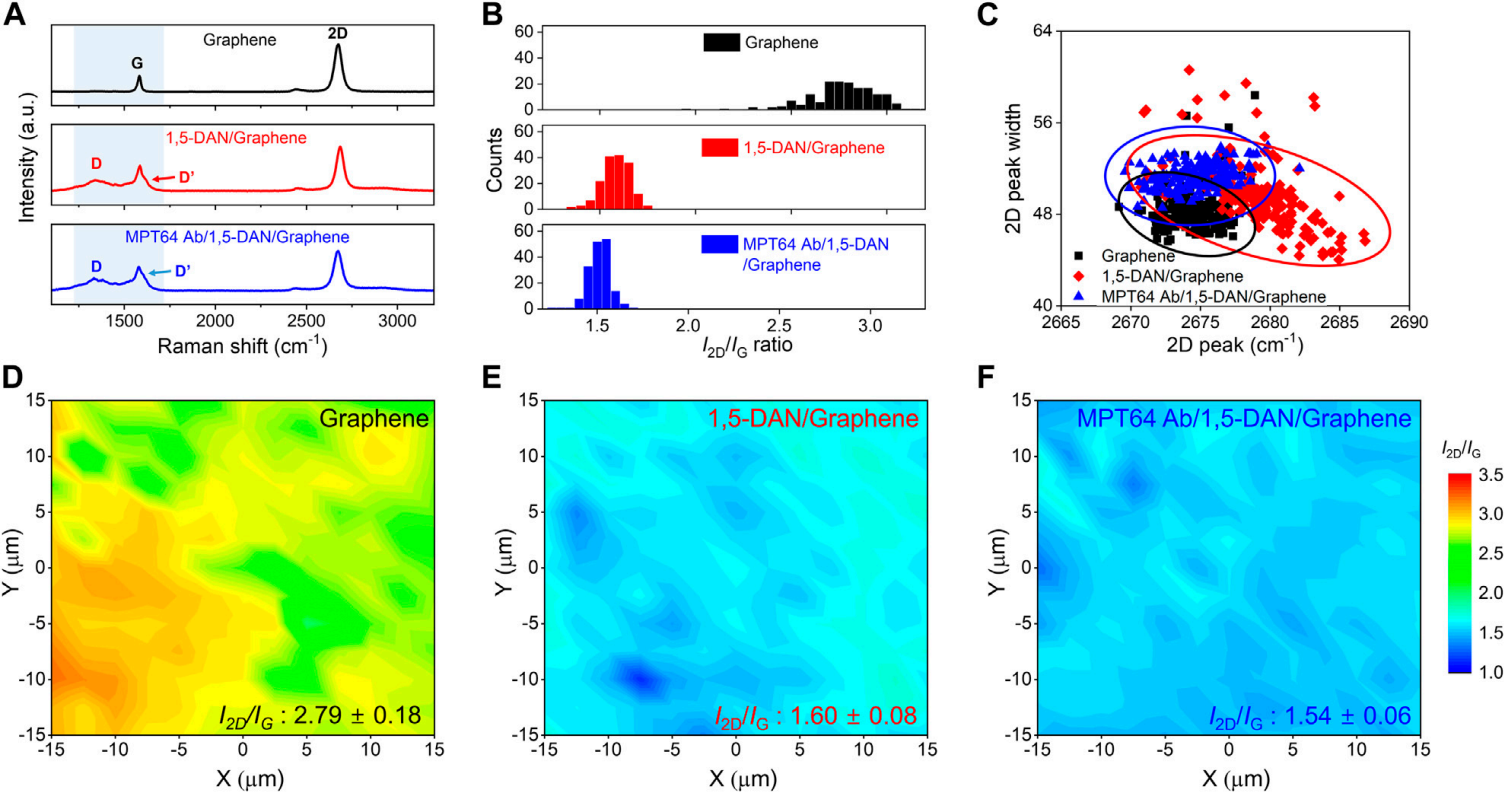
Raman spectroscopic characterization of surface properties of functionalized graphene **(A)**. Representative Raman spectra of pristine graphene (black line), 1,5-DAN-modified graphene (red line), and MTP64 Ab-conjugated graphene (blue line) **(B)**. Statistical distribution graph of the *I*_2D_/*I*_G_ ratio of pristine graphene, 1,5-DAN-modified graphene, and MTP64 Ab-conjugated graphene **(C)**. Scattering distribution analysis of the 2D peak (*x*-axis) and 2D peak width (*y*-axis) for pristine graphene (black squares), 1,5-DAN-modified graphene (red diamonds), and MTP64 Ab-conjugated graphene (blue triangles) **(D–F)**. Raman mapping images of the measured *I*_2D_/*I*_G_ ratios for pristine graphene, 1,5-DAN-modified graphene, and MTP64 Ab-conjugated graphene, respectively. (*N* = 196).

We collected Raman spectra (Figure 2) to confirm the successful chemical functionalization of the graphene surface with MTP64 Ab and 1,5-DNA. Figure 2A shows the Raman spectra of pristine graphene and the 1,5-DAN-modified graphene before and after the MTP64 Ab conjugation. The spectrum of pristine graphene typically exhibits three peaks at 1,353 cm^−1^ (D band), 1,583 cm^−1^ (G band), and 2,690 cm^−1^ (2D band). The D peak indicates the presence of defects in the graphene structure, the G peak corresponds to the carbon lattice vibration, and the 2D band corresponds to a second-order overtone of a different in-plane vibration (Ferrari and Basko, 2013).

In the Raman spectra of the pristine graphene (top, black line), the G and 2D bands are observed without the D peak. However, after the functionalization of the graphene surface with 1,5-DAN (middle, red line), noticeable peaks attributable to D and D’ appeared. The increase in intensity of the D peak is assumed to have occurred because of the presence of C–N, N–H, and C–H vibration modes from the 1,5-DAN on graphene in the range of 1,250–1,450 cm^−1^, near the D peak (Jackowska et al., 1995; Nguyen et al., 2016; Fraczyk et al., 2018). The D’ peak at ∼1,607 cm^−1^ also results from the skeletal vibration of the aromatic ring stretching from amines (Nguyen et al., 2016). Therefore, these bands indicate the resonance effect induced by 1,5-DAN on the graphene surface, confirming the successful functionalization of the graphene. After MTP64 Ab conjugation, the intensity of the D’ peak became ∼6.8% greater than that of the corresponding peaks in the spectrum of the 1,5-DAN functionalized graphene, which is attributable to the presence and functionality of amines, the main component of MTP64 Ab (Supplementary Figure S2) (Sypabekova et al., 2017).

Figure 2B shows statistical data for the 2D/G intensity ratio corresponding to each functionalization condition. The pristine graphene shows a 2D/G ratio of approximately 2.79. Given that a 2D/G ratio close to 3 indicates monolayer graphene, the sample was confirmed to be single-layer graphene (Cao et al., 2020). However, for the 1,5-DAN and MTP64 Ab/1,5-DAN, the 2D/G ratios are 1.60 and 1.52, respectively, because of the increase in the D’ peak intensity. Figure 2C shows the scattering distribution of the 2D peak widths measured at full-width at half-maximum (FWHM) as a function of the peak position. The pristine graphene shows an average 2D peak position at 2,674.31 cm^−1^ (black line). After functionalization with 1,5-DAN, the 2D peak position blue-shifted (p-type doping) by 4.96 cm^−1^ (from 2,674.31 to 2,679.27 cm^−1^, red line). We propose two hypotheses to explain these results. First, the 2D peak position can be upshifted because of overtone band modulation as the number of graphene layers increases (Calizo et al., 2007; Graf et al., 2007). That is, the benzene ring of 1,5-DAN can induce p-type characteristics in graphene through π–π stacking interactions on the graphene surface (as in the case of multilayer graphene). Second, from a charge transfer perspective, the highest occupied molecular orbital (HOMO) value of 1,5-DAN is −5.02 eV, which is lower than the Fermi level of graphene (−4.5 eV) (Nguyen et al., 2021; Mallela et al., 2023). Consequently, electrons from graphene transferred to 1,5-DAN, inducing a p-type doping effect in the graphene. By contrast, upon MTP64 Ab conjugation onto the 1,5-DAN-functionalized graphene, the 2D peak position red-shifted (n-type doping) by 4.68 cm^−1^ (from 2,679.27 to 2,674.59 cm^−1^, blue line). When MTP64 Ab is conjugated with 1,5-DAN-functionalized graphene, N atoms of the amine groups, the main component of MTP64 Ab, form sp^3^ bonds with the 1,5-DAN-functionalized graphene surface, leading to n-doping characteristics (Nguyen et al., 2021). This phenomenon aligns with the trends indicated in our scattering distribution data (Figure 2C; Supplementary Figure S3). Collectively, the Raman analysis results demonstrate the successful functionalization of MA and 1,5-DAN on the graphene surface. Figures 2D–F shows Raman mapping images of the 2D/G intensity ratio on the graphene surface. The 2D/G ratio values are consistently distributed in the measured region after functionalization with 1,5-DAN and GA, demonstrating uniform functionalization.

### 3.3 XPS- and UV–vis-based surface analyses

**FIGURE 3:**
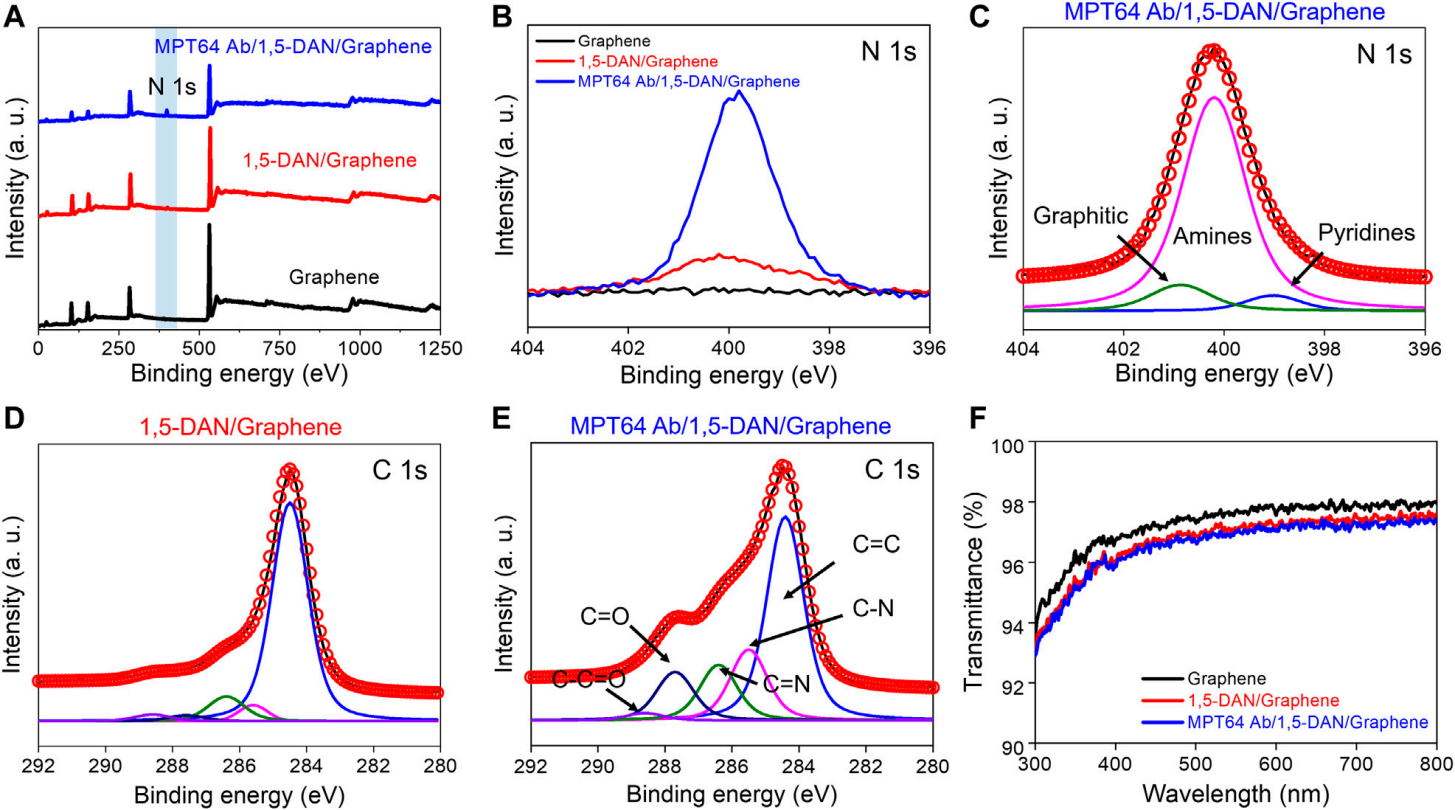
XPS analysis of the chemical binding on pristine graphene functionalized with 1,5-DAN and MTP64 Ab **(A)**. XPS survey peak of pristine graphene (black line), 1,5-DAN-modified graphene (red line), and MTP64 Ab-conjugated graphene (blue line). The filled blue box is the N 1s spectral region **(B)**. XPS spectra showing the N 1s peak of pristine graphene (black line), 1,5-DAN-modified graphene (red line), and MTP64 Ab-conjugated graphene (blue line) **(C)**. Deconvoluted XPS N 1s spectra for MTP64 Ab-conjugated graphene **(D,E)**. Deconvoluted XPS C 1s peak for 1,5-DAN-modified and MTP64 Ab-conjugated graphene, respectively **(F)**. Transmittance spectra of pristine graphene (black line), 1,5-DAN-modified graphene (red line), and MTP64 Ab-conjugated graphene (blue line) in the wavelength range 300–800 nm.

The quality of the graphene functionalized with 1,5-DAN and MTP64 Ab was evaluated by XPS analysis. Figure 3A shows XPS survey spectra of graphene (black line), 1,5-DAN-modified graphene (red line), and MTP64 Ab-conjugated graphene (blue line). N was not detected on the surface of the pristine graphene. The 1,5-DAN-treated graphene, however, contained a trace of N (∼1.46%). For the MTP64 Ab-conjugated graphene, the amount of N (∼5.96%) increased substantially (Figure 3B; Supplementary Table S2). Peak deconvolution of the N 1s peak was carried out to confirm the MTP64 Ab conjugation on the graphene surface (Figure 3A). The prominent peak in the N 1s spectrum in Figure 3C, which mainly appeared at ∼400 eV, indicates the presence of amines (Liu et al., 2013; Tai et al., 2014). This result shows that amine (–NH_2_) bonding affects the primary N composition, which contributes to the main components of the MTP64 Ab (Nguyen et al., 2021). We can, therefore, confirm the presence of MTP64 Ab on the 1,5-DAN-functionalized graphene surface on the basis of this peak. All these results strongly suggest that MTP64 Ab effectively conjugates to the surface of the 1,5-DAN-functionalized graphene.

Deconvoluted C 1s spectra of the 1,5-DAN- and MTP64 Ab/1,5-DAN-functionalized graphene are shown in Figures 3D, E, respectively. The C 1s peak is composed of five prominent peaks at 284.5, 285.5, 286.4, 287.7, and 288.6 eV, which correspond to C=C, C=N, C–N, C–OH, and C–C=O/C=O, respectively (Lee et al., 2019; Seo et al., 2020). The deconvolution results show that the spectrum of the MTP64 Ab-conjugated graphene had more functional-group peaks related to N and O than the spectrum of the 1,5-DAN-functionalized graphene (Figure 3D), indicating successful bonding of MTP64 Ab to the 1,5-DAN-modified graphene.

Figure 3F shows UV–Vis spectra that confirm the transparency of the functionalized graphene in the wavelength range 300–800 nm. Pristine graphene on a quartz substrate exhibited approximately 97.7% transparency at 550 nm, indicating single-layer graphene (Nair et al., 2008). In addition, after the functionalization with MTP64 Ab (red line) and 1,5-DAN (blue line), the transmittance decreased only negligibly (to ∼97%). These results show that the optical absorption of graphene films in the visible-light range is barely affected by the 1,5-DAN and MTP64 Ab functionalization.

### 3.4 Sensing performance of the TB-GFETs

**FIGURE 4:**
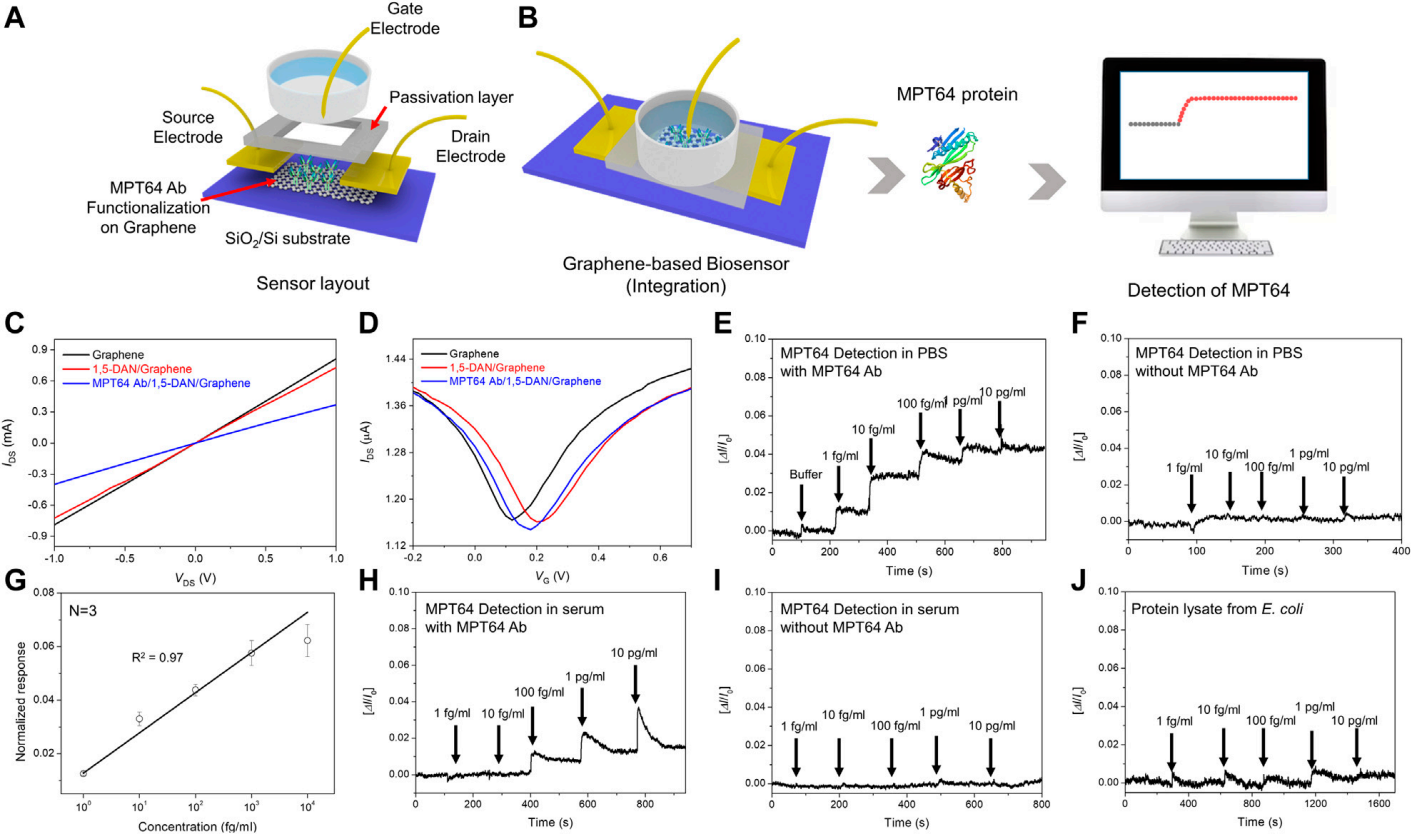
Sensing performance of the tuberculosis biosensor **(A)**. Schematic of the MTP64 Ab-functionalized GFET biosensor (TB-GFET) **(B)**. Schematics of detection of MPT64 using the TB-GFET with real-time detection results **(C)**. Current–voltage (*I−V*) characteristics of the graphene-based device after each functionalization process for MTP64 Ab modification **(D)**. Measurement of transfer curves of the TB-GFET sensor in steps of the antibody conjugation **(E)**. Real-time response of the TB-GFET toward MPT64 in PBS **(E)**. With MPT64 Ab and **(F)**. Without MPT64 Ab **(G)**. The calibration plot between sensor response and the MPT64 concentration. Real-time response of the TB-GFET toward MPT64 in serum **(H)**. With MPT64 Ab and **(I)**. Without MPT64 Ab. **(J)** selectivity test using total protein lysate from *E. coli*.

A schematic of the MTP64 Ab-functionalized GFET-based TB sensor (TB-GFET) developed in the present study is shown in Figure 4A The device configuration includes the functionalized graphene (sensing channel), gold electrodes (source/drain), and a sample well (reaction chamber) covered with a polymer passivation layer. Details of the fabrication process are provided in Figure 1A. As shown in Figures 4A, B MTP64 Ab-functionalized TB-GFET could detect the target MPT64 antigens by measuring the electrical signal changes via the difference in surface potentials in the channel. Because the functionalization molecules attach to the graphene surface, they can change the electrical properties of the graphene. Figure 4C shows current–voltage (*I–V*) curves over the range from −1.0 and 1.0 V for the graphene device before and after attachment of MTP64 Ab. After MTP64 Ab was immobilized onto the graphene channel surface, the slope (d*I*/d*V*) decreased. The variation in slope serves as an additional indicator of the successful attachment of MTP64 Ab because the adsorption of large molecules such as proteins onto the TB-GFET alters its electrical characteristics. In addition, the excellent linearity of the biosensors’ *I–V* curves, which indicates stable ohmic contact, enables quantitative analysis of the target analytes. We conducted measurements on the transfer curves of the TB-GFET following each modification process (Figure 4D). Following 1,5-DAN functionalization, a distinct positive shift was observed, attributed to the p-doping effect of the pyrene group. However, upon immobilization of the antibody, the transfer curve exhibited a negative shift.

Transient responses of the TB-GFET were measured as a function of the MPT64 concentration in PBS (Figure 4E; Supplementary Figure S4). The sensing responses were collected by recording the drain–source current (*I*) with different MPT64 concentrations. The electrical signal changed from the baseline (PBS) to the new state upon introduction of MPT64 samples with different concentrations. The sensing signal was determined by assessing the normalized change in current, represented as [Δ*I*/*I*_0_], where *I*_0_ represents the initial current and *I* is the current measured after achieving stability following the change in MPT64 concentration. Because MPT64 has an isoelectric point of 4.6, the MPT64 will be negatively charged at pH = 7.4, resulting in hole accumulation in the graphene channel. Thus, the MPT64 should behave as an electrochemical gate with a negative potential, resulting in an increase in the *I* for the p-type TB-GFET. The response of the MPT64 Ab-conjugated TB-GFET gradually increases with increasing MPT64 concentration, reaching saturation at approximately 10 pg/mL. The detection limit for this device is ∼1 fg/mL, indicating that highly sensitive detection of MPT64 is possible. However, when we conducted the measurement using an unfunctionalized GFET, no reactivity toward MPT64 was observed (Figure 4F). The results of this control experiment indicate that the MPT64 Ab is critical for specific binding with the MPT64. Figure 4G shows the calibration plot of the antigen, which exhibits a linear relationship with the corresponding MPT64 concentration in the range of 0.001–10 pg/mL, with an *R* ^2^ value of 0.97.

In clinical settings, MTB diagnosis commonly relies on serum samples. Consequently, we evaluated the FET sensor’s responsiveness to antigens present in serum. To assess its applicability in practical field, we measured the FET sensor’s response to MPT64 in diluted serum. The findings demonstrated the sensor’s ability to detect MPT64 at concentrations as low as 100 fg/mL (Figure 4H). This suggests that the TB-GFET sensor can identify antigens in clinical samples without requiring preprocessing. In contrast, measurements using an un-functionalized GFET showed no reactivity toward MPT64 (Figure 4I). Furthermore, the TB-GFET showed no response to proteins from *E. coli*, indicating that our TB-GFET sensor specifically detects the MPT64 antigen protein from MTB (Figure 4J).

### 3.5 Comparison of MPT64 detection using different methods

**FIGURE 5:**
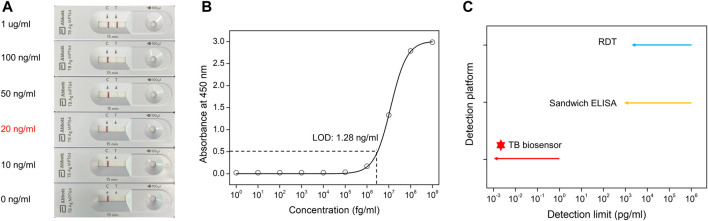
Sensitivity comparison with different sensing platforms for detecting MPT64 **(A)**. Rapid diagnostic test (RDT) kit detection results for MPT64 antigen proteins **(B)**. The binding affinity of the MPT64 Ab to MPT64 antigen proteins, as measured by ELISA **(C)**. Comparison of our TB-GFET and other bio-sensing methods (RDT and ELISA) for MPT64 detection.

To demonstrate the testing efficiency of the TB-GFET technique compared with that of conventional testing methods for detecting MPT64 antigen, we compared the results obtained using the TG-GFET technique with those obtained using a rapid diagnostic test (RDT) kit and an enzyme-linked immunosorbent assay (ELISA) kit The response using the commercialized RDT method results revealed that the limit of detection (LOD) of the MPT64 is ∼20 ng/mL (Figure 5A). Notably, the signals became faint as the concentration decreased and not signal was detected at concentrations less than 10 ng/mL. We also measured the sensitivity of the MPT64 antigen using ELISA; the LOD was calculated to be 1.28 ng/mL (Figure 5B). These two methods can detect MPT64 at ng/mL concentration levels; however, the sensitivity requires further improvement for early diagnosis without requiring an incubation process. The sensitivity limitation observed in both RDT and ELISA for MPT64 detection could be addressed using the TB-GFET, which achieved an LOD of 1 fg/mL for direct MPT64 detection in PBS (Figure 5C). Furthermore, when examining previous results that utilized electrochemical sensors for detecting MTB, it is evident that platforms employing electrochemical measurement methods, including the FET sensor, have lower LOD compared to ELISA and RDT methods (Supplementary Table S3).

## 4 Conclusion

We demonstrated a graphene-based biosensor—specifically, a field-effect transistor—for detecting MPT64, a protein secreted by the MTB complex species. The channel region of the TB-GFET was chemically modified using 1,5-DAN and GA linkers to covalently immobilize MTP64 Ab onto the graphene surface. The graphene surfaces with linker molecules and those conjugated with MTP64 Ab were characterized via a collective set of experiments that included AFM, Raman spectroscopy, XPS, and *I–V* measurements to confirm the successful functionalization of the TB-GFET. The TB-GFET successfully detected MPT64 antigen proteins at concentrations as low as 1 fg/mL, implying that the device enables the highly sensitive detection of MPT64 without a pre-incubation step. However, GFET biosensors cannot replace ELISA or RDT due to their small dynamic range; rather, it can have its specific uses in potentially aiding in early detection or management of relapses in infectious diseases. In the future, research and development should focus on developing purpose-built, miniaturized biosensor systems. This innovative sensing approach has the potential to revolutionize point-of-care diagnostics and open up opportunities for the diagnosis of various emerging infectious diseases. Therefore, the developed highly sensitive TB-GFET provides a promising platform for POC detection of TB and can also be applied to the detection of other emerging infectious diseases.

## Data Availability

The original contributions presented in the study are included in the article/Supplementary Material, further inquiries can be directed to the corresponding authors.
