# Supplementary material for: Ultrasensitive biosensing platform for Mycobacterium tuberculosis detection based on functionalized graphene devices
Source: Front Bioeng Biotechnol. 2023 Dec 20;11:1313494. doi: 10.3389/fbioe.2023.1313494 (PMC10765604; doi:10.3389/fbioe.2023.1313494)
Supplement: Supplementary file 1 [file DataSheet1.docx]

Supplementary Material

Ultrasensitive Biosensing Platform for *Mycobacterium tuberculosis* Detection Based on Functionalized Graphene Devices

Giwan Seo^1,2,†^, Geonhee Lee^3,†^, Wooyoung Kim^1,2^, Inyoung An^1,2^, Myungwoo Choi^4^, Sojeong Jang^3^, Yeon-Joon Park^5^, Jeong-O Lee^3*^, Donghwi Cho^3*^ and Edmond Changkyun Park^1,2*^

^1^ Research Center for Bioconvergence Analysis, Korea Basic Science Institute, Cheongju, 28119, Republic of Korea

^2^ Critical Diseases Diagnostics Convergence Research Center, Korea Research Institute of Bioscience and Biotechnology, Daejeon 34141, Republic of Korea

^3^ Advanced Materials Division, Korea Research Institute of Chemical Technology, Daejeon, 34114, Republic of Korea

^4^ Department of Materials Science and Engineering, Korea University, Seoul 02841, Republic of Korea

^5^ Department of Laboratory Medicine, The Catholic University of Korea, Seoul St. Mary's Hospital, Seoul, 06591, Republic of Korea

^†^ These authors contributed equally to this work.

*** Correspondence:**Jeong-O Lee (jolee@krict.re.kr)

Donghwi Cho (roy.cho@krict.re.kr)

Edmond Changkyun Park (edpark@kbsi.re.kr)


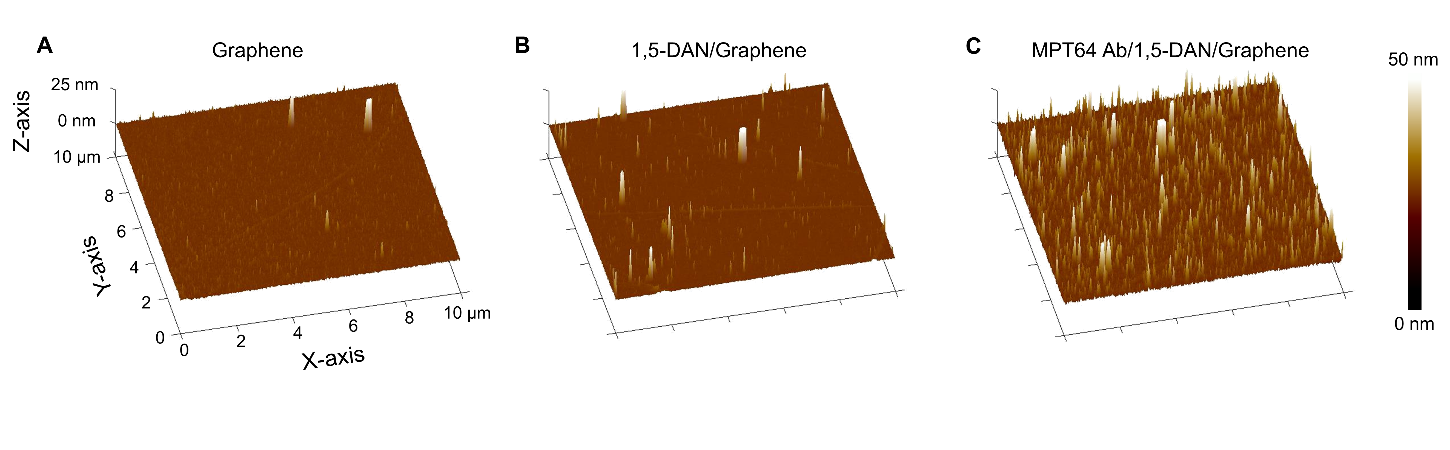


**Supplementary Figure 1.** AFM 3D image of **a.**Graphene, **B.**1,5-Dan/Graphene and **C.** MPT64 Ab/1,5-DAN/Graphene from **Figure 1B-D**.


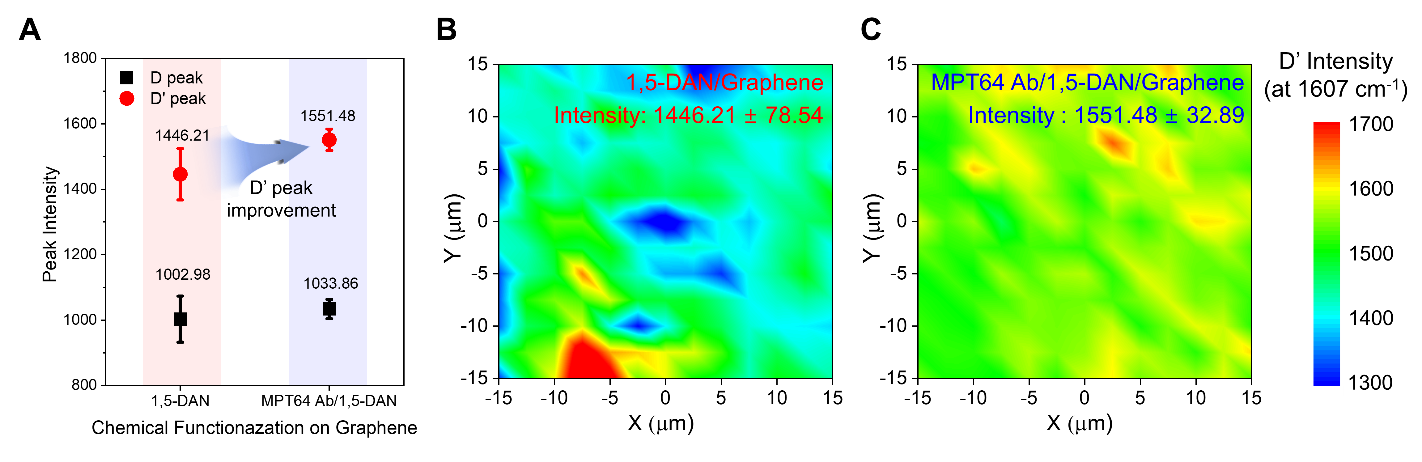


**Supplementary Figure 2.** **A.** Averaged D and D' peak positions of 1,5-DAN/graphene (black squares) and MAb/1,5-DAN/graphene (red circles). **B–C.** D' position mapping images of 1,5-DAN/graphene and MPT64 Ab/1,5-DAN/graphene, respectively. (*N* = 196)


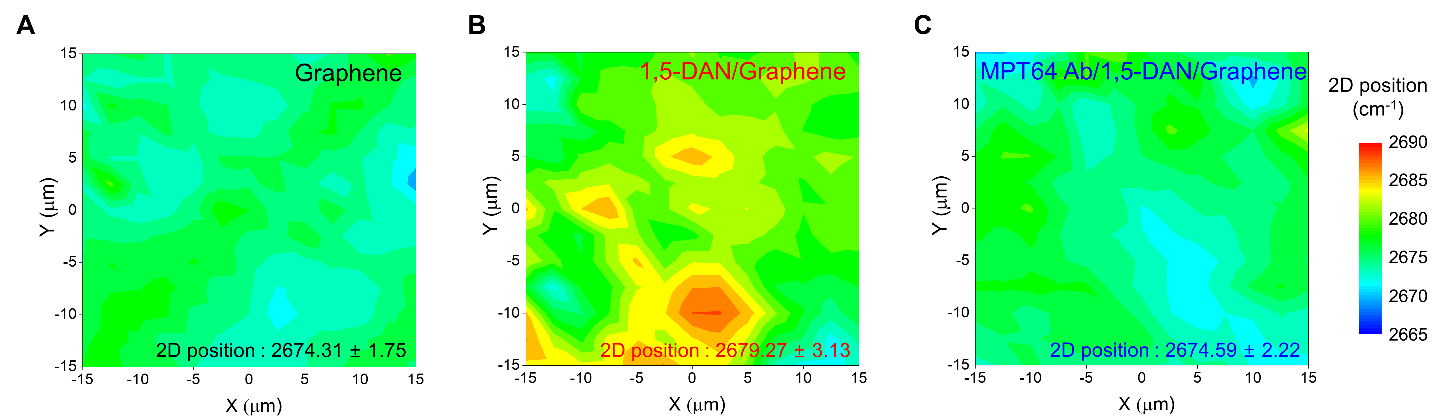


**Supplementary Figure 3.** 2D position mapping image of **A**. Graphene, **B**. 1,5-DAN/graphene, and **C** .MPT64 Ab/1,5-DAN/graphene. (*N* = 196)


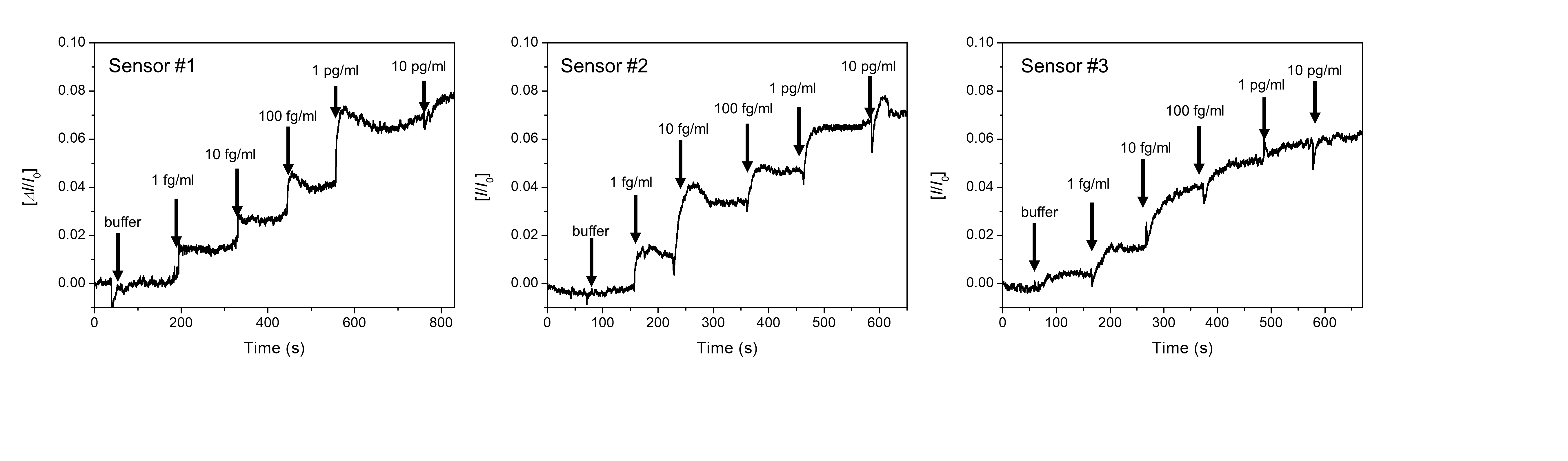


**Supplementary Figure 4.** Real-time signal response for MPT64 detection using three TB-GFETs.

| **Material** | **Brand** | **Purpose** |
| --- | --- | --- |
| Polymethyl methacrylate (PMMA) C4 | Microchem | Graphene transfer  (Supporting layer) |
| CE-100 Cu etchant | Transene | Graphene transfer (Cu etching) |
| 300 MIF developer | Microchem | Photolithography |
| AZ5214 photoresist | Microchem | Photolithography |
| 1.5-Diaminonaphthalene (1,5-DAN) | Sigma-Aldrich | Functionalization on Graphene |
| Glutaraldehyde (GA) | Sigma-Aldrich | Functionalization on Graphene |
| MPT 64 antibody (MPT64 Ab) | Abcam | Receptor |
| M. *tuberculosis* protein (MPT 64) | Abcam | Detection target material |
| Carbonate-bicarbonate butter | Sigma-Aldrich | Immunosorbent assay |
| Tween 20 | Sigma-Aldrich | Immunosorbent assay |
| Bovine serum albumin (BSA) | Abcam | Immunosorbent assay |
| HRP-conjugated anti-rabbit IgG | Cell Signaling Technology | Immunosorbent assay |
| Stabilized chromogen (TMB) | ThermoFisher Scienfitic | Immunosorbent assay |

**Supplementary Table 1.** Reagents and materials.

| **%** | **Graphene** | **1,5-DAN/graphene** | **MPT64 Ab/1,5-DAN/graphene** |
| --- | --- | --- | --- |
| C | 48.19 | 51.98 | 56.55 |
| O | 51.81 | 46.56 | 37.48 |
| N | 0 | 1.46 | 5.96 |
| Total | 100 | 100 | 100 |

**Supplementary Table 2.** Atomic composition analysis of C, O, and N from **Figure 3A**

| **Type of sensor** | **Bioreceptor** | **Analyte** | **LOD** | **Ref.** |
| --- | --- | --- | --- | --- |
| voltammetry | biotin-capture probe | MPT64 | 67.6 fg/mL | (Li et al., 2022) |
| voltammetry | aptamer | MPT64 | 10 fg/mL | (Li et al., 2018) |
| voltammetry | aptamer | MPT64 | 0.9 fg/mL | (Thakur et al., 2017) |
| amperometry | aptamer | MPT64/CFP10 | 1.68/1.82 ng/mL | (Yunus et al., 2022) |
| FET | antibody | MPT64 | 1 fg/mL | This work |

**Supplementary Table 3.** Comparison table showing the MTB detection performance of our FET biosensor and other published ones

# Reference

Li, N., Huang, X., Sun, D., Yu, W., Tan, W., Luo, Z., et al. (2018). Dual-aptamer-based voltammetric biosensor for the Mycobacterium tuberculosis antigen MPT64 by using a gold electrode modified with a peroxidase loaded composite consisting of gold nanoparticles and a Zr(IV)/terephthalate metal-organic framework. *Microchim. Acta* 185, 1–7. doi: 10.1007/s00604-018-3081-2.

Li, Y., Peng, D., Guo, S., Yang, B., Zhou, J., Zhou, J., et al. (2022). Aptasensor for Mycobacterium tuberculosis antigen MPT64 detection using anthraquinone derivative confined in ordered mesoporous carbon as a new redox nanoprobe. *Bioelectrochemistry* 147, 108209. doi: 10.1016/j.bioelechem.2022.108209.

Thakur, H., Kaur, N., Sabherwal, P., Sareen, D., and Prabhakar, N. (2017). Aptamer based voltammetric biosensor for the detection of Mycobacterium tuberculosis antigen MPT64. *Microchim. Acta* 184, 1915–1922. doi: 10.1007/s00604-017-2174-7.

Yunus, M. H., Yusof, N. A., Abdullah, J., Sulaiman, Y., Ahmad Raston, N. H., and Md Noor, S. S. (2022). Simultaneous Amperometric Aptasensor Based on Diazonium Grafted Screen-Printed Carbon Electrode for Detection of CFP10 and MPT64 Biomarkers for Early Tuberculosis Diagnosis. *Biosensors* 12, 996. doi: 10.3390/bios12110996.
